# A Comparative Evaluation of Marginal Bone Loss Around Dental Implants Using Slow- and Medium-Speed Drilling Without Irrigation Versus High-Speed Drilling With Irrigation: An In Vivo Study

**DOI:** 10.7759/cureus.84730

**Published:** 2025-05-24

**Authors:** Shitij Srivastava, Shubham K Srivastava, Abhinav Shekhar, Anshuman Chaturvedi, Debajyoti Sarkar

**Affiliations:** 1 Department of Prosthodontics, Sardar Patel Post Graduate Institute of Dental and Medical Sciences, Lucknow, IND

**Keywords:** alveolar bone loss, dental implants, maxilla, randomized controlled trial, surgical flaps

## Abstract

Aim and objectives

In this study, we aimed to compare the impacts of slow-speed (50 rpm) drilling without irrigation and medium-speed (300 rpm) drilling without irrigation with high-speed (800 rpm) drilling with irrigation on marginal bone loss around the dental implant.

Material and methods

This study was conducted and reported following the CONSORT (Consolidated Standards of Reporting Trials) guidelines. This randomized controlled trial utilized a within-subjects design, wherein each participant received three implants in the unilateral maxillary posterior region. A minimum of 24 patients (72 implants, three per patient) was proposed to ensure adequate statistical power and equal allocation across all intervention arms. Each implant site was prepared using a distinct drilling protocol: slow-speed (50 rpm, without irrigation), medium-speed (300 rpm, without irrigation), and high-speed (800 rpm, with irrigation). Randomization of implant sites was performed using the Research Randomizer website to reduce bias. The 50- and 300-rpm sites were designated as the case group, while the 800-rpm site served as the control. All procedures followed a standardized two-stage surgical protocol, with implants placed 1 mm subcrestally and flaps closed using 3-0 silk sutures. Drilling torque was maintained at 45 Ncm. Periapical radiographs were obtained immediately postoperatively and at six months using standardized settings (70 kV, 8 mA, 0.2 seconds, 30 cm of focus distance) with a customized XCP positioning system. Marginal bone levels were measured on mesial and distal aspects using ImageJ software. A single-blind design was implemented to ensure that the radiologist assessing bone loss was unaware of the group allocations.

Results

The study compared the marginal bone loss among two case groups and a control group based on drilling speeds: 50 rpm without irrigation (subgroup A), 300 rpm without irrigation (subgroup B), and 800 rpm with irrigation (group C), respectively. Mesial and distal bone loss was significantly lower in subgroup A (mesial: 0.383 ± 0.018 mm; distal: 0.403 ± 0.018 mm) compared to subgroup B (mesial: 0.434 ± 0.014 mm; distal: 0.440 ± 0.014 mm) and group C (mesial: 0.477 ± 0.013 mm; distal: 0.491 ± 0.013 mm). Statistical analysis revealed significant differences between groups (ANOVA: p < 0.001). Pairwise comparisons confirmed the order of marginal bone loss as group C > subgroup B > subgroup A.

Conclusion

The study demonstrated that slow-speed drilling without irrigation resulted in significantly lower marginal bone loss compared to medium-speed drilling without irrigation and high-speed drilling with irrigation. These findings suggest that slow-speed drilling may offer a clinically advantageous alternative for implant site preparation, promoting better osseointegration and reducing the risk of implant failure.

## Introduction

Success in restorative dentistry has been the cornerstone for an ever-challenging solution for tooth replacement, namely, dental implants [1]. One of the foremost concerns in terms of implants is marginal bone loss (MBL), irrespective of success rates, which may undermine implant stability and compromise overall treatment success. MBL tends to occur in the early phase of post-implant positioning and can be influenced by many factors such as surgical procedure, design of the implant, and biological effects [2]. Thus, an understanding of MBL from its underlying factors becomes extremely essential in the quest to maximize the longevity of implants and better clinical outcomes.

Endosseous implants comprise a steadfast and efficacious method for rehabilitating the oral cavity, with treatment success largely depending on the advancement of bone healing [3]. Bone healing after implant placement may occur either via repair or regeneration, though the latter appears to be required for long-term osseointegration [4]. Successful osseointegration requires rigid adherence to certain surgical and biological protocols. Among these, properly preparing the implant site is paramount for optimal healing. Excessive heat during osteotomy can negatively affect bone healing, predisposing it to problems such as hyperemia, necrosis, fibrosis, and osteocytic degeneration, which amplify osteoclastic activity while impairing osseointegration [5].

The event cascades to a biological process being triggered by implant placement, as well as the host tissue, which will respond to the presence of this foreign material. The human body can exhibit four central responses to the presence of foreign material: rejection, dissolution, resorption, or demarcation [6-7]. Demarcation is considered a protective mechanism where the body isolates a foreign object that cannot be resorbed or dissolved, usually leading to fibrous encapsulation. But if the biocompatible material is placed in an environment free from infection and micro-movements over a prolonged period, it tends to get encapsulated with bone, thus forming a robust bone-to-implant interface; this process, where the implant would get accepted by the bones, is called osseointegration [8]. Most successfully osseointegrated implants show long-term clinical success due to stable activity in remodeling bone. Any deviation from balance between bone apposition and resorption will result in MBL and thus threaten the longevity of the implants [9].

Early MBL is a non-infectious remodeling that occurs during the first year of implant placement and has a multifactorial etiology. Surgical aspects that operate toward MBL are inadequate crestal width, malposition of the implant, excessive cortical compressions, and thermal injuries during site preparation. Other prosthetic factors included in this etiology are the implant-abutment connection type, location of microgap, frequency of abutment disconnections, abutment height, residual cement, and early loading [10-13]. Early MBL is said to be an adaptive response of peri-implant bone to all these factors and is a crucial predictor for the success of the implants in the long run. If studies have shown MBL greater than 0.44 mm within six months of prosthetic loading, it could be interpreted that the implant is at a greater risk of progressive peri-implant bone loss [14].

The depth of implant placement and its relation to the implant-abutment junction are known to be significant factors influencing early MBL. A technique known as "platform switching" has been shown to reduce early MBL significantly; it consists of using an abutment narrower than the implant neck to horizontally displace the microgap from the bone crest [15-16]. However, the conventional drilling techniques performed at high speeds create excessive heat that may cause thermal necrosis of the bone, impairing osseointegration and thus enhancing MBL. To reduce this probability, copious irrigation and intermittent drilling are employed [17-18].

## Materials and methods

Ethical approval

This study received ethical approval from the Institutional Ethical Committee (PROSTHO/06/222336/IEC) and was prospectively registered with the Clinical Trials Registry - India (CTRI/2023/08/056139).

Research design

**Figure 1:**
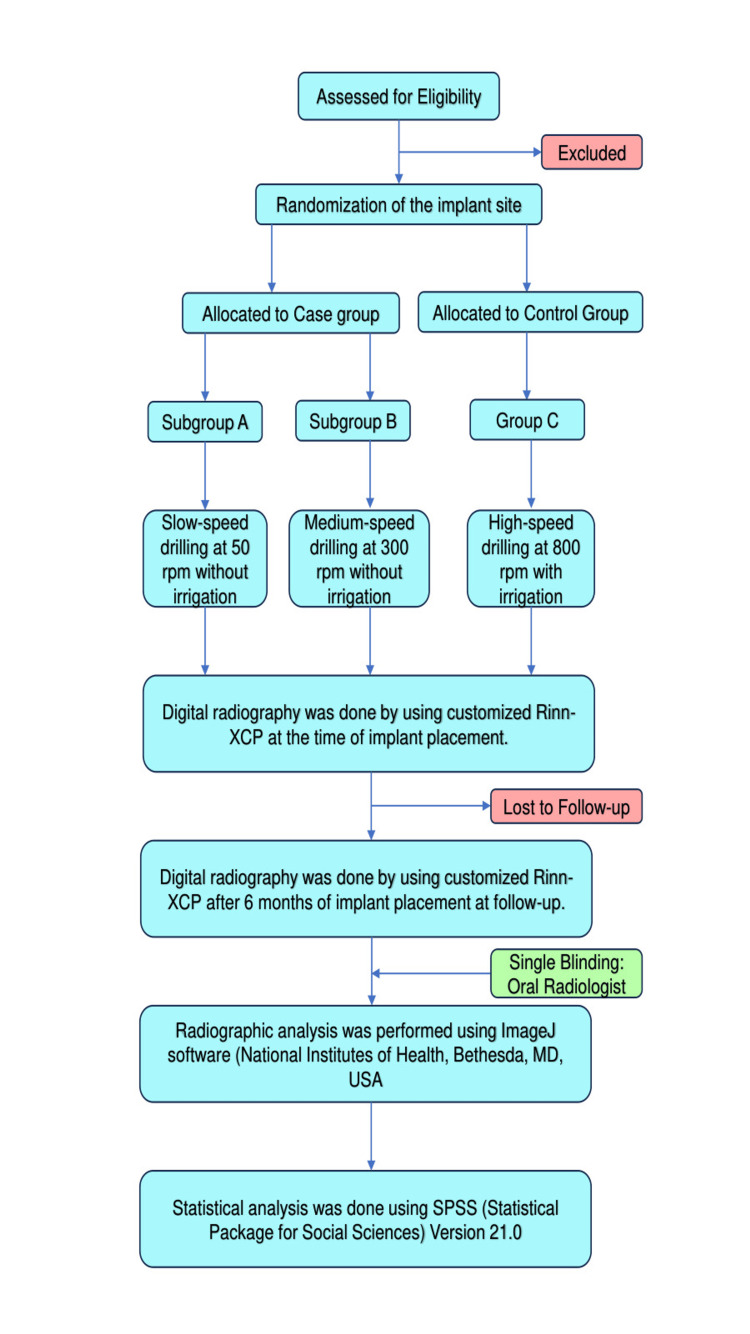
CONSORT flowchart of the study CONSORT, Consolidated Standards of Reporting Trials

This study was conducted and reported following the CONSORT (Consolidated Standards of Reporting Trials) guidelines. The study was a randomized controlled trial with a within-patient comparison to evaluate the effects of different drilling speeds on MBL in dental implants (Figure 1).

Sample size estimation

The sample size for this study was determined based on a previous investigation, which reported a mean MBL of 0.49 ± 0.42 mm using the low-speed drilling technique and 0.44 ± 0.35 mm using the conventional-speed drilling [19]. The calculation was performed using the formula recommended by Charan and Biswas [20]:

\[ n \;=\; \frac{\bigl(\tfrac{r + 1}{r}\bigr)\,\bigl[\mathrm{SD}^2 \times \bigl(Z_{\beta} + Z_{\alpha/2}\bigr)^2\bigr]}{d^2} \]

where r is the number of interventions (3), Z_α/2_ is 1.96 (for 95% confidence), Z_β _is 0.84 (for 80% power), SD is 0.07 (difference in standard deviation), and d is 0.05 (difference in means). Substituting these values yielded n = 20.5, which was rounded to 21 and further adjusted to account for a 10% potential data loss. Thus, a minimum of 24 patients (72 implants, three per patient) was proposed to ensure adequate statistical power and equal allocation across all intervention arms.

Randomization and group allocation

In this study, randomizing implant sites in the maxillary posterior region involved assigning two implants to the case group and one to the control group for each patient. The randomization process began by labeling the three implant sites as implant site 1, implant site 2, and implant site 3 for each patient. A randomization website (https://www.randomizer.org) was then used to assign each implant to either the subject or control group. For instance, two of the implants would be randomly assigned to the subject group (50-rpm slow-speed drilling without irrigation; 300-rpm medium-speed drilling without irrigation), while the remaining implant would be allocated to the control group (800-rpm high-speed drilling with irrigation). This randomization ensured that each patient had an unbiased allocation of the implant site, minimizing any potential selection or placement bias.

Blinding

In this study, the evaluation of marginal bone levels was conducted at two key time points: immediately after surgery and after a six-month healing period. Radiographs were taken at both time points, and the analysis of these images was performed using ImageJ software (National Institutes of Health, Bethesda, MD, USA). The focus of the analysis was on measuring the MBL around the implants, which was quantified at the mesial and distal aspects of each implant.

To minimize bias in the assessment process, a single-blinding approach was employed. The oral radiologist, who performed the image analysis, was blinded to the group allocation of the implants. This meant that the radiologist was not aware of which implants belonged to the case group or the control group during the evaluation process. This single-blind approach was crucial for reducing the potential for bias in interpreting the radiographs and ensured that the analysis remained objective and unbiased throughout the study. By maintaining blinding, the study aimed to provide reliable and valid results regarding the impact of different drilling protocols on MBL around dental implants.

Sample selection

Participants aged 18-65 years with at least three missing teeth in the maxillary posterior region, adequate alveolar ridge width (>5 mm), and sufficient bone quality and quantity were included, provided they were systemically healthy and consented to participate. Exclusion criteria included NSAID allergy, history of periodontitis, chemotherapy or radiotherapy, bisphosphonate therapy, tobacco use, need for sinus augmentation, pregnancy, systemic conditions affecting healing, mucosal diseases, recent tooth extraction (within 4 months), and parafunctional habits. Patients were withdrawn if they discontinued participation or failed to report for prosthetic rehabilitation or follow-up visits.

Methodology

This randomized controlled trial employed a within-subjects design, where each participant received three implants in the unilateral maxillary posterior region, with each implant placed using a different drilling speed: slow-speed drilling at 50 rpm without irrigation, medium-speed drilling at 300 rpm without irrigation, and high-speed drilling at 800 rpm with irrigation. Implant sites were randomly allocated using Research Randomizer, with two sites assigned to the case group (50 rpm and 300 rpm) and one to the control group (800 rpm with irrigation). The study followed a standardized surgical protocol to ensure consistency and minimize bias.

All participants received MIS Seven implants (MIS Implants Technologies Ltd., Misgav, Israel), which are tapered, self-tapping, screw-type titanium implants featuring a sandblasted, large-grit, acid-etched (SLA) surface designed to enhance primary stability. Implant selection was tailored to each patient based on preoperative assessment of bone volume and quality. Local anesthesia was administered, and sulcular and crestal incisions were made to raise a full-thickness flap. Drilling was performed at 600 rpm initially for both groups, with subsequent speeds adjusted based on the group allocation. In contrast, in the control group, initial drilling was done at 600 rpm, followed by consequent drilling at 800 rpm with irrigation using physiological saline. A torque of 45 Ncm was maintained during osteotomy preparation, and implants were placed 1 mm subcrestally using a two-stage technique. Wound closure was performed with 3-0 silk sutures. All surgeries were conducted by a single surgeon to standardize procedures.

Radiographic analysis was conducted at two time points: immediately postoperatively and after six months. Images were obtained using standardized periapical techniques and consistent radiographic settings (70 kV, 8 mA, 0.2 s, 30 cm of focus distance). A customized XCP bite block ensured consistency. MBL was assessed mesially and distally using ImageJ software, with a single-blind approach to minimize observer bias. The radiologist was blinded to implant group allocation to ensure objective analysis.

Measurements of marginal bone loss

**Figure 2:**
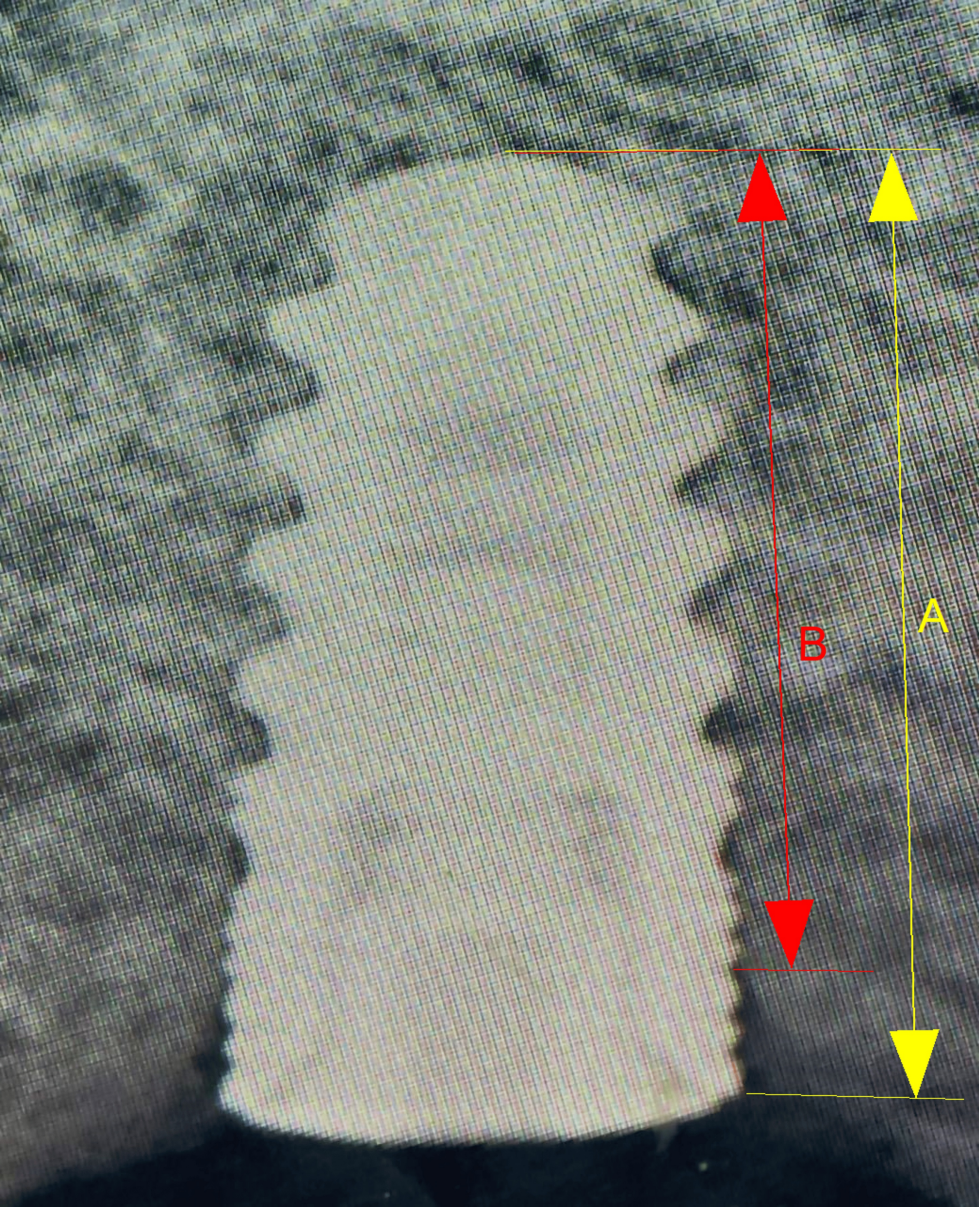
MBL assessment through dental radiograph A. Distance between the implant apex and the implant platform. B. Distance between the implant apex and first bone-to-implant contact. ImageJ software (National Institutes of Health, Bethesda, MD, USA) MBL, marginal bone loss

MBL was quantified by measuring the vertical distance from the implant platform - used as a stable reference point - to the first visible bone‐to‐implant contact on both mesial and distal aspects, using high‐resolution, calibrated periapical radiographs. Baseline distances were recorded immediately after implant placement, and follow‐up measurements were obtained at six months to capture bone remodeling dynamics (Figure 2).

For each implant, site-specific MBL was calculated as the difference between follow-up and baseline distances:

\[ \mathrm{MBL}_{\mathrm{site}} = D_{\mathrm{follow\text{-}up}} - D_{\mathrm{baseline}} \]

The mean MBL per implant was determined by averaging the mesial and distal values:

\[ \mathrm{MBL}_{\mathrm{implant}} = \frac{\mathrm{MBL}_{\mathrm{mesial}} + \mathrm{MBL}_{\mathrm{distal}}}{2} \]

## Results

Out of the 24 patients (three implant sites in each) enrolled, drilling was done at 50 rpm without irrigation at 24 implant sites (33.3%) and at 300 rpm without irrigation at 24 implant sites (33.3%). These were grouped as cases and individually categorized as subgroups A and B, respectively. In the remaining 24 implant sites (33.3%), drilling was done at 800 rpm with irrigation; these were categorized as group C, i.e., controls (Table 1).

**Table 1:** Group-wise distribution of patients (maxillary posterior edentulism)

Group		Drilling speed	No. of implant sites	Percentage
Cases	Subgroup A	50 rpm without irrigation	24	66.6
	Subgroup B	300 rpm without irrigation	24	
Controls	Group C	800 rpm with irrigation	24	33.3

Among implant sites categorized as cases (slow-speed and medium-speed drilling without irrigation), the range of mesial and distal bone loss was 0.35-0.46 mm and 0.37-0.46 mm, respectively; the mean mesial bone loss was 0.409±0.030 mm, and the mean distal bone loss was 0.421±0.025 mm (Table 2).

**Table 2:** Mesial and distal bone loss in cases (subgroups A and B)

Location of bone loss	No. of implant sites	Minimum	Maximum	Mean	SD
Mesial	48	0.35	0.46	0.409	0.030
Distal	48	0.37	0.46	0.421	0.025

Among cases, mean distal bone loss (0.421±0.025 mm) was found to be higher than that of mesial bone loss (0.409±0.030 mm). On comparing the difference between distal and mesial bone loss among cases, this difference was found to be statistically significant (p<0.001) (Table 3).

**Table 3:** Difference in mesial and distal bone loss among cases Statistical analysis was performed using a paired t-test.

S. No.	Location of bone loss	Mean	SD
1	Mesial	0.409	0.030
2	Distal	0.421	0.025
Difference in mesial and distal bone loss		0.013	0.014
Statistical significance		t=6.224; p<0.001	

Among implant sites that underwent drilling at 50 rpm without irrigation, i.e., subgroup A, the drilling range of mesial and distal bone loss was found to be 0.35-0.41 mm and 0.37-0.43 mm, respectively. Mean mesial bone loss among these patients was 0.383±0.018 mm, and distal bone loss was 0.403±0.018 mm. Distal bone loss was found to be higher than that of mesial bone loss (Table 4).

**Table 4:** Mesial and distal bone loss in subgroup A

Location of bone loss	No. of implant sites	Minimum	Maximum	Mean	SD
Mesial	24	0.35	0.41	0.383	0.018
Distal	24	0.37	0.43	0.403	0.018

Among subgroup A difference between distal bone loss (0.403±0.018 mm) and mesial bone loss (0.383±0.018 mm) was found to be statistically significant (Table 5).

**Table 5:** Difference in mesial and distal bone loss in subgroup A (cases with 50-rpm drilling speed) Statistical analysis was performed using a paired t-test.

S. No.	Location of bone loss	Mean	SD
1	Mesial	0.383	0.018
2	Distal	0.403	0.018
Difference in mesial and distal bone loss		0.019	0.009
Statistical significance		t=10.112; p<0.001	

The range of mesial and distal bone loss among implant sites undergoing drilling at 300 rpm (Subgroup B) without irrigation was 0.41-0.46 mm and 0.42-0.46 mm. Mean mesial and distal bone loss among Subgroup B patients was 0.434±0.014 mm and 0.440±0.014 mm, respectively (Table 6).

**Table 6:** Mesial and distal bone loss in subgroup B

Location of bone loss	No. of implant sites	Minimum	Maximum	Mean	SD
Mesial	24	0.41	0.46	0.434	0.014
Distal	24	0.42	0.46	0.440	0.014

Among subgroup B implant sites, distal bone loss (0.440±0.014 mm) was higher as compared to the mesial bone loss (0.434±0.014 mm). The difference in mesial and distal bone loss among subgroup B implant sites was not found to be statistically significant (Table 7).

**Table 7:** Difference in mesial and distal bone loss in subgroup B (cases with 300-rpm drilling speed) Statistical analysis was performed using a paired t-test.

S. no.	Location of bone loss	Mean	SD
1	Mesial	0.434	0.014
2	Distal	0.440	0.014
Difference in mesial and distal bone loss		0.006	0.015
Statistical significance		t=1.941; p=0.065	

Among implant sites undergoing high-speed drilling (800 rpm with irrigation) that had been categorized as controls (group C), the range of mesial and distal bone loss was 0.46-0.50 mm and 0.47-0.51 mm, respectively (Table 8).

**Table 8:** Mesial and distal bone loss in controls (group C)

Location of bone loss	No. of implant sites	Minimum	Maximum	Mean	SD
Mesial	24	0.46	0.50	0.477	0.013
Distal	24	0.47	0.51	0.491	0.013

Among group C implant sites, mean distal bone loss (0.491±0.013 mm) was found to be higher than that of mesial bone loss (0.477±0.013 mm). The difference in mesial and distal bone loss among controls (0.014±0.016 mm) was found to be statistically significant (Table 9).

**Table 9:** Difference in mesial and distal bone loss in group C, i.e., controls Values are presented as mean ± SD. Statistical analysis was performed using a paired t-test.

S. no.	Location of bone loss	Mean	SD
1	Mesial	0.477	0.013
2	Distal	0.491	0.013
Difference in mesial and distal bone loss		0.014	0.016
Statistical significance		t=4.331; p<0.001	

When the bone loss between cases and controls was compared, the mesial bone loss in cases (0.409±0.030 mm) was found to be significantly less than that in controls (0.477±0.013 mm) (Table 10). Similarly, distal bone loss in cases (0.421±0.025 mm) was found to be significantly less than that in controls (0.491±0.013 mm) (Table 10).

**Table 10:** Comparison of mesial and distal bone loss between cases and controls Values are presented as mean ± SD. Statistical analysis was performed using an independent samples t-test.

Location of bone loss	Cases (N=48)		Controls (N=24)		Statistical significance	
	Mean	SD	Mean	SD	t	p
Mesial	0.409	0.030	0.477	0.013	10.569	<0.001
Distal	0.421	0.025	0.491	0.013	12.876	<0.001

**Figure 3:**
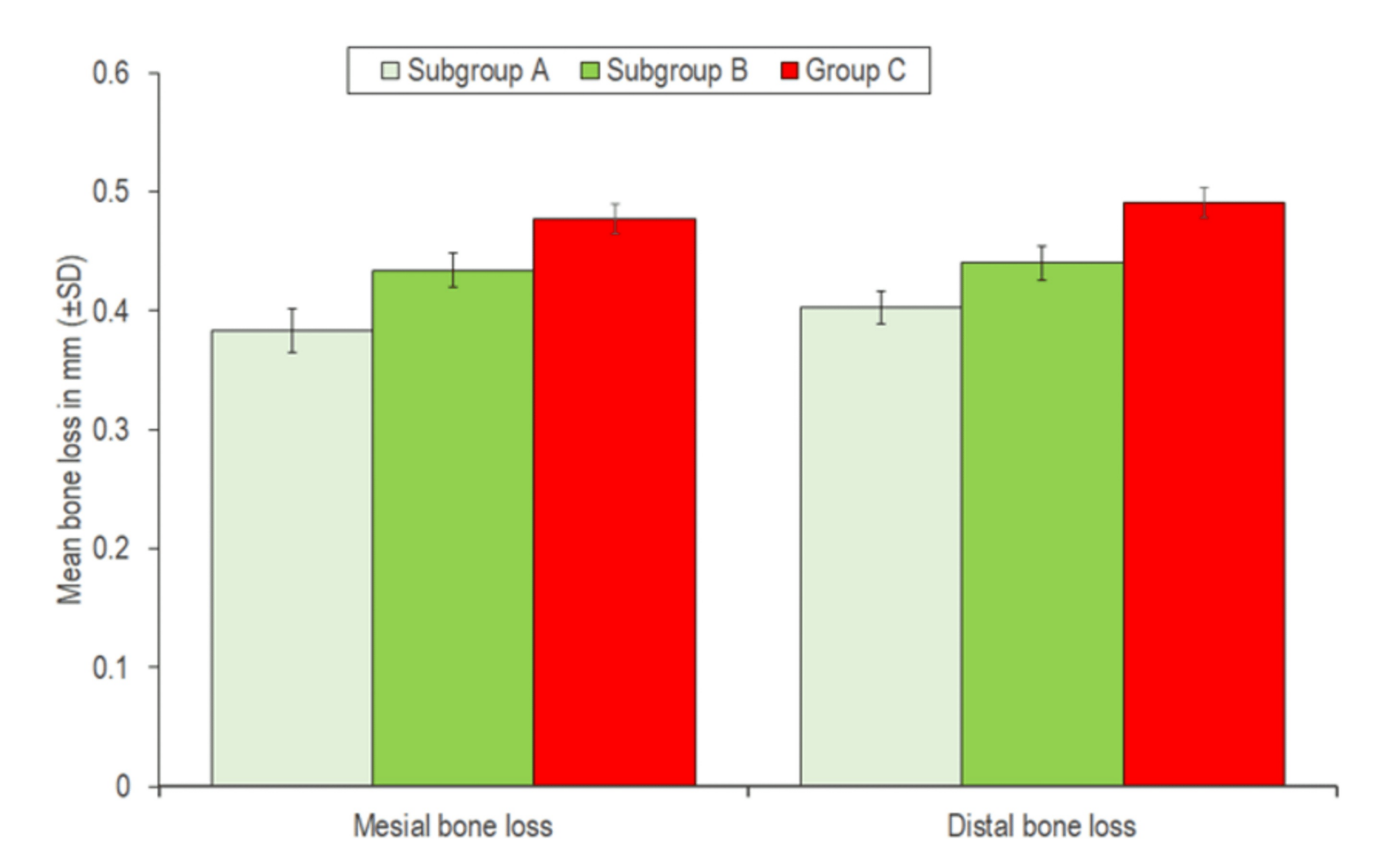
Comparison of mesial and distal bone loss in patients with different drilling speeds (subgroup A, subgroup B, and group C)

Mesial bone loss in subgroup A (0.383±0.018 mm) was minimum, followed by that in subgroup B (0.434±0.014 mm), and the maximum in group C (0.477±0.013 mm); this difference was found to be statistically significant (Table 11; Figure 3). Similarly, distal bone loss in subgroup A (0.403±0.018 mm) was minimal, followed by that in subgroup B (0.440±0.014 mm), and the maximum in group C (0.491±0.013 mm); this difference was found to be statistically significant (Table 11; Figure 3).

**Table 11:** Comparison of mesial and distal bone loss in patients with different drilling speeds Values are presented as mean ± SD. Statistical analysis was performed using one-way ANOVA.

	N	Mesial bone loss		Distal bone loss	
		Mean	SD	Mean	SD
Subgroup A cases (50 rpm)	24	0.383	0.018	0.403	0.018
Subgroup B cases (300 rpm)	24	0.434	0.014	0.440	0.014
Group C (controls) (800 rpm)	24	0.477	0.013	0.491	0.013
Statistical significance (ANOVA)		F=230.661; p<0.001		F=205.572; p<0.001	

On exploring the between-group differences of mesial bone loss, the maximum difference was found between subgroup A and group C (0.094±0.004), followed by subgroup A and subgroup B (0.051±0.004), and the minimum between subgroup B and group C (0.043±0.004); all the between-group differences were found to be statistically significant. Therefore, the order of mesial bone loss was group C > subgroup B > subgroup A (Table 12). Similarly, exploring the between-group differences in distal bone loss, a maximum difference was found between subgroup A and group C (0.088±0.004), followed by subgroup B and group C (0.051±0.004), and a minimum between subgroup A and subgroup B (0.038±0.004); all the between-group differences were found to be statistically significant. Therefore, the order of distal bone loss was group C > subgroup B > subgroup A (Table 12).

**Table 12:** Between-group differences in mesial and distal bone loss Values are presented as mean difference ± standard error (SE) for each pairwise comparison. Statistical significance was determined using post-hoc tests following one-way ANOVA.

Group	Mesial bone loss			Distal bone loss		
	Mean	SE	p	Mean	SE	p
Subgroup A vs. subgroup B	-0.051	0.004	<0.001	-0.038	0.004	<0.001
Subgroup A vs. group C	-0.094	0.004	<0.001	-0.088	0.004	<0.001
Subgroup B vs. group C	-0.043	0.004	<0.001	-0.051	0.004	<0.001

## Discussion

This study evaluated the impact of different drilling speeds - 50 rpm (subgroup A), 300 rpm (subgroup B), and 800 rpm (group C) - on MBL around dental implants. The findings demonstrated that lower drilling speeds, particularly 50 rpm without irrigation, were associated with significantly reduced mesial and distal bone loss compared to medium- and high-speed drilling protocols. These results affirm the hypothesis that slow-speed drilling mitigates thermal and mechanical trauma to peri-implant bone, promoting improved bone preservation and potentially enhancing implant success.

Subgroup A (50 rpm without irrigation) exhibited the lowest MBL (mesial: 0.383 ± 0.018 mm; distal: 0.403 ± 0.018 mm), significantly outperforming both the medium-speed group (subgroup B) and the high-speed control group (group C). These results align with existing literature emphasizing the benefits of low-speed drilling in reducing thermal damage and preserving bone vitality. Slow drilling minimizes thermal necrosis and enhances osseointegration by reducing peak temperatures during osteotomy preparation [19].

Interestingly, the absence of irrigation in subgroup A did not compromise bone preservation, suggesting that the lower mechanical energy generated at 50 rpm may be inherently protective. This finding has significant clinical implications, particularly for patients with compromised bone quality, where minimizing trauma is critical. Bone temperatures exceeding 47°C can cause irreversible cell damage; thus, a slower drilling speed may be inherently advantageous in avoiding such thresholds without the need for irrigation [21].

Subgroup B (300 rpm without irrigation) demonstrated moderately increased bone loss (mesial: 0.434 ± 0.014 mm; distal: 0.440 ± 0.014 mm), indicating that while medium-speed drilling may offer some preservation benefits, it is less effective than slower protocols. The lack of statistically significant difference between mesial and distal bone loss in this group (p = 0.065) may reflect a threshold at which mechanical and thermal stresses begin to outweigh the protective benefits of slower rotation, especially in the absence of irrigation.

Group C (800 rpm with irrigation), the standard high-speed protocol, showed the highest bone loss values (mesial: 0.477 ± 0.013 mm; distal: 0.491 ± 0.013 mm). These findings highlight that irrigation alone may not sufficiently counteract the thermal effects of rapid drilling. Despite being a widely used technique, high-speed drilling, even with saline cooling, may lead to greater marginal bone resorption, corroborating prior studies that implicate rapid drilling in thermal injury and mechanical overload to bone.

The ANOVA test confirmed statistically significant differences in bone loss among the three groups (p < 0.001), and post-hoc comparisons particularly emphasized the superiority of the 50-rpm group over the 800-rpm control group. Low-speed drilling without irrigation yielded MBL levels comparable to conventional methods but with additional safety benefits for thermally sensitive cases [22].

However, contrasting evidence exists. Drilling speed had minimal influence on bone temperature and healing outcomes in an animal model; however, it is noteworthy that the 50-rpm group exhibited higher cortical temperatures despite demonstrating favorable primary stability [23]. Similarly, increased drilling speeds did not significantly impact bone viability unless combined with deeper drilling [24]. These discrepancies may be attributed to differences in methodology, including in vivo versus in vitro conditions, irrigation techniques, drilling depth, and bone quality.

Despite these conflicting reports, the current study strengthens the case for low-speed drilling as a viable and potentially superior protocol for preserving marginal bone. It is important to note that the benefits of slow-speed drilling may be more pronounced in specific clinical scenarios, such as patients with poor bone density or when irrigation is not feasible. Nevertheless, the increased operative time and potential for greater tactile feedback required during slow drilling should also be considered when tailoring protocols to individual patient needs.

Limitations and future directions

The sample size was limited, and the lack of histological evaluation precludes definitive conclusions regarding osseointegration quality. Additionally, the results are specific to drilling protocols without variations in drill geometry, bone density, or operator technique. Future studies incorporating temperature measurement during drilling, histomorphometric analysis, and long-term implant stability assessments would further elucidate the biological impact of drilling speed. Clinical trials across diverse patient populations are also essential to validate these findings in broader contexts.

## Conclusions

Within the limitations of this study, it can be concluded that drilling speed plays a critical role in the preservation of marginal bone around dental implants. The use of a slow-speed drilling protocol at 50 rpm without irrigation resulted in significantly less mesial and distal bone loss compared to medium-speed (300 rpm) and high-speed (800 rpm with irrigation) protocols. These findings suggest that reduced rotational speed may minimize thermal and mechanical trauma during osteotomy preparation, thereby enhancing bone preservation and potentially improving implant outcomes. Clinicians may consider adopting slow-speed drilling techniques, particularly in patients with compromised bone quality, as a means to optimize peri-implant bone health. Further clinical studies with larger sample sizes and long-term follow-up are recommended to validate these findings and assess their impact on implant survival and success rates.
